# Insights on Calcium-Dependent Protein Kinases (CPKs) Signaling for Abiotic Stress Tolerance in Plants

**DOI:** 10.3390/ijms20215298

**Published:** 2019-10-24

**Authors:** Rana Muhammad Atif, Luqman Shahid, Muhammad Waqas, Babar Ali, Muhammad Abdul Rehman Rashid, Farrukh Azeem, Muhammad Amjad Nawaz, Shabir Hussain Wani, Gyuhwa Chung

**Affiliations:** 1 Department of Plant Breeding and Genetics, University of Agriculture, Faisalabad 38000, Pakistan; luqmanshahid73@gmail.com (L.S.); bhuttawaqas@ymail.com (M.W.); babar1292ali@gmail.com (B.A.); rashidpbg@hotmail.com (M.A.R.R.); 2 Center for Advanced Studies in Agriculture and Food Security, University of Agriculture, Faisalabad 38040, Pakistan; 3 Industrial Crops Research Institute, Yunnan Academy of Agricultural Sciences, Kunming 650200, China; 4 Department of Bioinformatics and Biotechnology, Government College University, Faisalabad 38040, Pakistan; azeuaf@hotmail.com; 5 Education Scientific Center of Nanotechnology, Far Eastern Federal University, 690950 Vladivostok, Russia; amjad_ucauos@yahoo.com; 6 Mountain Research Centre for Field Crops, Sher-e-Kashmir University of Agricultural Sciences and Technology of Kashmir, Srinagar 190001, India; shabirhussainwani@gmail.com; 7 Department of Biotechnology, Chonnam National University, Chonnam 59626, Korea

**Keywords:** calcium-dependent protein kinases, calcium signaling, ABA, drought, salinity

## Abstract

Abiotic stresses are the major limiting factors influencing the growth and productivity of plants species. To combat these stresses, plants can modify numerous physiological, biochemical, and molecular processes through cellular and subcellular signaling pathways. Calcium-dependent protein kinases (CDPKs or CPKs) are the unique and key calcium-binding proteins, which act as a sensor for the increase and decrease in the calcium (Ca) concentrations. These Ca flux signals are decrypted and interpreted into the phosphorylation events, which are crucial for signal transduction processes. Several functional and expression studies of different CPKs and their encoding genes validated their versatile role for abiotic stress tolerance in plants. CPKs are indispensable for modulating abiotic stress tolerance through activation and regulation of several genes, transcription factors, enzymes, and ion channels. CPKs have been involved in supporting plant adaptation under drought, salinity, and heat and cold stress environments. Diverse functions of plant CPKs have been reported against various abiotic stresses in numerous research studies. In this review, we have described the evaluated functions of plant CPKs against various abiotic stresses and their role in stress response signaling pathways.

## 1. Introduction

Plants have several adaptive features to cope with biotic and abiotic stresses under challenging environmental situations. Plants respond to these stresses by inducing the expression of stress-responsive genes through a complex signaling pathway. The expression of these stress-responsive genes is induced upon changes in calcium ion (Ca^2+^) concentrations, due to various biotic and abiotic stimuli [1,2], which enable plant adaptations in a wide range of stressed environments.

Calcium (Ca) as a ubiquitous secondary messenger regulates the stress signaling mechanism in plants. Changes in Ca^2+^ concentration are sensed by several calcium-binding proteins, especially calcium-dependent protein kinases [3]. The calcium-dependent abiotic and biotic stress signaling mechanisms are most commonly dominated by calcium-dependent protein kinases, which play a pivotal role in the regulation of plant responsiveness to salt, drought, and cold and heat stresses as well as other environmental factors. Ca^2+^ is involved in abscisic acid (ABA)-dependent biotic and abiotic stress signals in various plant species [4,5]. The calcium-dependent protein kinases phosphorylate the ABA-responsive element-binding factors (ABFs). ABA regulation by Ca^2+^ is associated with plant defense systems through induction of antioxidants [6], including reactive oxygen species (ROS) [2], and other enzymes like superoxide dismutase (SOD), catalase 3 (CAT3), ascorbate peroxidase (APX), glutathione peroxidase (GPX), and glutathione reductase (GR) [6,7]. It is also involved in the induction of some nonenzymatic antioxidants like ascorbic acid, α-tocopherol, carotenoids, and glutathione and controls multiple abiotic stress response processes [6,8,9,10]. This review will provide insight into the role of calcium-dependent protein kinases (CPKs) in abiotic stress tolerance in different plant species.

## 2. CPK Enzymes and Related Kinases

Several calcium-binding protein families have been identified in plants, which are potentially involved in the regulation of calcium-dependent abiotic stress response mechanisms. These Ca^2+^ sensors decode and transmit complex information, present in the form of calcium signal, to the phosphorylation events and regulate stress-responsive genes through protein interactions [11]. These Ca^2+^ signal-decoding groups include calcium-dependent protein kinases (CDPKs or CPKs), calmodulins (CaMs), calmodulin-like protein kinases (CMLs), calcineurin β-like proteins (CBLs), and Ca^2+^/calmodulin-dependent protein kinase (CCaMK) [12,13]. Among all these kinases, CPKs, CMLs, and CBLs have only been discovered in plants and some protozoans, while CaMs are highly conserved among all eukaryotes [11,14]. CaMs, CBLs, and CMLs are small proteins that function as calcium signal communicators through binding to downstream effectors (EFs) [15,16]. CaMs evolved from CMLs, which are considered as the most primitive calcium-binding proteins [13]. Among all these, CPKs were identified in plants as well as green algae, oomycetes, and in some protozoans [17], but they are not present in animals. CPKs, through direct binding with Ca^2+^, have a predominant regulatory role for the Ca-sensing protein families [17].

## 3. CPK Family in Plants

CPKs are considered as the versatile player for the regulation of abiotic stress management in plants [17]. In 1984, the very first plant CPKs were identified in *Pisum sativum* [18]. These proteins were initially purified from soybeans in 1987. A CPK encoding gene was cloned from *Arabidopsis thaliana* in 1991, which opened new ways for CPK gene cloning in several other plant species [11,19,20]. The presence of CPKs in almost all parts of the plant demonstrates that these kinases have a high potential for regulating various signal transduction pathways and have a significant influence on plant growth and development [17,21,22,23].

### 3.1. CPK Distribution and Localization in Plants

CPKs show a widespread distribution in different plant species. The whole-genome sequencing of plant species (e.g., *Arabidopsis* [24]) enables researchers to conduct genome-wide identifications of variable CPK encoding genes. These studies identified 34 CPK-encoding genes in the genome of *Arabidopsis thaliana*, 20 in *Triticum aestivum* (wheat), and 31 in *Oryza sativa* (rice) [20,25,26]. *Solanum lycopersicum* (tomato), which is a model plant of the *Solanaceae* family, has 29 CPK-encoding genes [27]. Genome-wide exploration of some other plants such as *Zea mays* (maize), *Hordeum vulgare* (barley), *Cucumis melo* (melon), *Populus trichocarpa* (poplar), *Gossypium raimondii* (cotton), *Manihot esculenta* (cassava), and *Vitis vinifera* (grapevine) revealed the presence of 40, 28, 18, 30, 41, 27, and 19 CPK-encoding genes, respectively [28,29,30,31,32,33,34] (Table 1). Mostly, CPK-encoding genes are expressed in leaves, meristems, roots, and flowers, while some are expressed only in specific tissues [23,35,36].

Similarly, CPKs are also found in pollens, embryonic cells, guard cells, xylem, and meristem [36]. These Ca-dependent functional proteins are involved in biological functioning in cellular and subcellular compartments. Numerous CPKs of *Arabidopsis* are membrane-localized. It is considered that the myristylation causes CPKs to target the membrane [62]. This cellular and subcellular localization indicates a significant role of CPKs in several signaling transduction pathways under stress stimuli.

### 3.2. CPK Domain Organization and Calcium Ion Signal Decryption

On account of specific abiotic stress stimuli, the plant activates distinct physiological and biochemical response pathways. These stimuli are perceived by some protein and nonprotein elements. Protein elements include enzymes, transcription factors, and disparate receptors, while nonproteins comprise some secondary messengers such as calcium ion cyclic nucleotides, hydrogen ions, lipids, and active oxygen species [17,63]. Among them, Ca is a crucial secondary messenger involved in the signal transduction in all eukaryotes. It regulates the cell polarity and is essential for the regulation of stress-responsive cellular processes, cell morphogenesis, as well as plant growth and development [3,11,64,65]. These calcium signals are recognized by several protein kinases (CPKs), which regulate the response of downstream factors.

**Figure 1:**
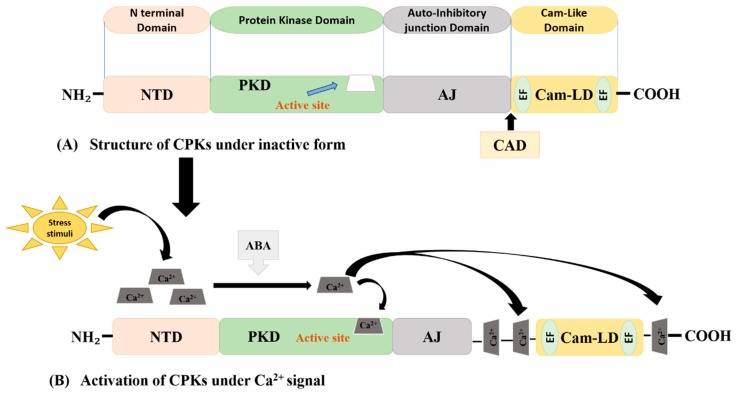
Structure and activation process of plant CPKs. (**A**) CPK domain structure under the inactive state, (**B**) activation of CPKs after the binding of Ca^2+^ to the active site of the protein kinase domain (PKD), the autoinhibitory junction (AJ), and calmodulin-like domain (CaM-like domain, CaM-LD).

The CPK-encoding protein commonly has four functional domains, viz., calcium-binding domain (CBD), N terminus variable domain (NTD), protein kinase domain (PKD), and autoinhibitory junction (AJ), but many CPKs also contain an amino-terminal domain with varying sequence lengths, which is a source of functional diversity in the CPK family [62]. Sometimes, the C-terminus variable domain (CTD) also considered as a distinct domain instead of NTD. Different plant species contain varying numbers of CPK genes that are functionally important. The CBD contains four loops where calcium ions directly bind, called EF-hands, and are 20 amino acids in length [20,66,67,68]. The PKD domain has a characteristic serine/threonine phosphorylation site, which responds during regulation of CBD and AJ through Ca signals [68,69]. Among the number of CPK proteins, the majority of them have a myristylation site upstream from their N-terminal variable domain, showing that no CPKs appear in the form of membrane integral proteins [23]. The N-terminus of CPKs has a greater percentage of proline, glutamine, serine, and threonine (PEST) sequences, which carry out swift proteolytic degradation. There is an auto-inhibitory domain adjacent to the conserved domains, having a pseudo-substrate domain activity, and can cause inhibition of the regulatory pathways [68]. The variation in the length of CPK genes is due to the NTD, CT domain, and EF hand of the calcium-binding domain. Ca^2+^ through binding with the EF-hand motif, carries out the phosphorylation of the CPK substrate by removing autoinhibition of kinase activity [22,70]. The highly conserved calmodulin-like domain regulates all the activities of the CPKs by binding the four Ca^2+^ ions to four EF hands at its downstream end. Proteomics of most of the CPKs show that the autophosphorylation of proteins at serine and threonine through a calcium-dependent manner regulate the kinase activity (Figure 1).

CPKs are monomolecular Ca-signaling protein kinases that regulate protein phosphorylation. In response to extrinsic and intrinsic cues, the variation in Ca^2+^ concentration, also called “Ca^2+^ signatures”, is recognized, interpreted, and transduced to the downstream toolkit by a group of Ca^2+^-binding proteins. Phosphorylation events cause the activation of CPKs.

### 3.3. Functional Characterization of Plant CPKs

CPKs are differentially involved in diverse and indispensable functions in various plant species. CPKs show their role against biotic and abiotic stress tolerance upon interaction with specific calcium signals. With respect to abiotic stresses, CPKs are involved in drought [71], salinity [72], and heat [73] and cold [74] stress response signaling by regulating the ABA-responsive transcriptional factors and ion channel regulation [75]. Some *Arabidopsis* CPKs (e.g., *CPK13*) are also involved in potassium ion (K^+^) channel regulation and other ion transportation in guard cells [11]. CPKs are also a major participant for providing pathogen-related immunity to plants. In several plant species, CPKs enhance the resistance against fungal elicitors [1,76,77], bacterial invasions [78], and many other pathogen-related diseases [60,79]. Some CPKs are involved in the regulation of the jasmonic acid (JA)-dependent pathway during insect and plant interaction and indirectly regulate plant resistance against insects [80]. The crucial role of CPKs have also been reported in various growth and developmental processes in plants. CPK-encoding genes (*AtCPK28*) in *Arabidopsis* play a positive role in stem elongation and contribute to secondary growth by interacting with the gibberellic acid (GA) pathway [81,82]. Similarly, some CPKs regulate pollen tube growth [83], latex biosynthesis [55,84], higher biomass accumulation [85], wounding and herbivory attack [80,86], germination and seedling growth [87], early maturity [88,89], pigmentation and fruit development [90], and several other metabolic and developmental pathways [91]. Still, the role and functionality of various CPK-encoding genes against biotic and abiotic stresses are veiled.

## 4. Role of CPKs in Abiotic Stress Tolerance

CPKs are recognized as a key Ca sensor group of protein kinase, having a multigene family in the whole plant kingdom [55,92]. The functions of these CPKs are completely dependent on Ca^2+^ signatures. Most of CPK functionality has been identified only in vitro, which is why only specific stress response-associated functions are known [93]. CPKs are not only involved in ion channel regulation but also respond to multiple stress-related pathways through interactions with other distant transcription factors through phosphorylation. Several loss-of-function and gain-of-function studies have confirmed the role of CPKs in abiotic stress tolerance. The cytosolic Ca^2+^ concentration fluxes, induced by various environmental stresses, viz., heat [47], cold [94] light [95], drought [96,97], salt [72,98], and osmotic [99] and pathogen-related factors [100], activate the plant’s transcriptional and metabolic activities [101]. Expression analyses and genome-wide studies have discovered the CPKs transcript activity, protein, and substrate recognition in different plant parts [93]. CPKs are also involved in the ABA-dependent abiotic stress signaling in various plant species. Several CPK genes are involved in the regulation of ABA signaling pathways in plants. Transient gene expression analyses in protoplasts of maize show that *CPK11* (closely related to *AtCPK4* and *AtCPK11*) acts upstream of mitogen-activated proteins (MPK5) and is required for the activation of defense functions and antioxidant enzyme activity by regulating the expression of MPK5 genes. Similarly, *CPK11* induced by hydrogen peroxide (H_2_O_2_) regulates and controls the activity of SOD and APX production induced by the ABA signaling pathway [102,103]. CPK activity confirmed by global expression analyses, shows that several CPK members are expressed differentially under varying ABA, salinity, drought, and heat and cold levels [93]. The change in the expression of CPK genes indicates the role of CPKs in plant adaptation against abiotic stress environments.

### 4.1. CPK-Mediated Drought Response Signaling

Drought stress is a major destructive factor affecting plant growth and development. It decreases water potential in plants as a result, where ABA accumulation controls the opening and closing of stomata, which leads to a lower photosynthetic activity [104]. It decreases the biomass and grain yield in plants. Under drought, plants adopt several conformational changes in the cell. These include ABA-dependent stomatal movement through regulation of guard cells, osmotic adjustments through the accumulation of osmolytes, regulating the oxidative damage by ROS homeostasis, and so on [93,105]. Changes in cytosolic Ca^2+^ concentrations due to water deficiency initiates CPK activity, resulting in the release of ABA in the cell [97]. ABA induces the injection of a calcium chelator (i.e., 1,2-bis (2-aminophenoxy) ethane-*N*,*N*,*N*′,*N*′-tetra acetic acid; BAPTA), into the guard cell, which causes the closing of the stomata and, eventually, control of the transpiration process. Several plant CPKs are involved in drought stress-response mechanisms through an ABA-dependent manner. The CPK-encoding gene (*CPK10*) of *Arabidopsis* and an identified interacting heat shock protein (HSP1) lead to a drought-sensitive genotype. *CPK10* T-DNA insertional mutants show sensitivity to drought stress as compared to the wild types. *AtCPK9* and *AtCPK10* are involved in Ca^2+^-dependent ABA-mediated stomatal regulation through interaction with *AtCPK33* [106]. The light-induced *Arabidopsis* encoding gene (CPK13) is involved in inhibiting stomatal opening and contributes to the drought stress responsiveness [11]. Some drought-responsive CPKs also have some associated functions. In rice, for example, *OsCPK9* controls both drought stress tolerance and spikelet fertility through an ABA-dependent manner. Results of overexpression of *OsCPK9* (*OsCPK9*-OX) induces stomatal closure through osmotic adjustment and increases the pollen viability and spikelet fertility under polyethylene glycol (PEG-6000)-induced drought stress [71]. Another CPK-encoding gene from the wild grapevine (*CPK20*) acts as a regulator for drought and its associated with heat/cold responsive pathways. Expression of these genes studied in transgenic *Arabidopsis* reveals that *VaCPK20* overexpression exhibits a high level of tolerance to drought and cold stress through regulation of stress responder genes, viz., ABA-responsive element binding factor 3 (ABF3) or sodium/hydrogen exchanger 1 (NHX1), and cold regulator gene (*COR47*) [107]. While a CPK-encoding gene of broad bean (*VfCPK1*) reported being highly expressed in leaf epidermal peels, it is not considered a tissue-specific gene and is only expressed under drought stress [108]. This CPK-encoding gene shows no relationship with both high (37 °C) and low (4 °C) temperatures. The increase in the number of transcripts of *VfCPK1* under drought stress only plays a role in the up-regulation of ABA-responsive genes and other kinases that are involved in the signal transduction pathway [108].

Some CPKs are involved in the regulation of antioxidant production and osmolyte homeostasis to combat drought stress. *AtCPK8* regulates the movement of the stomatal guard cell and H_2_O_2_ homeostasis in response to cellular Ca^2+^. An *Arabidopsis* T-DNA insertion mutant of *CPK8* was found to be more sensitive to drought stress as compared to the wild-type plant, which reveals their drought response functionality [97]. CPKs phosphorylate some interactional proteins and perform interactive functioning in plants. Under drought stress, *AtCPK8* with an interacting protein CAT3 controls the Ca^2+^-dependent ABA-mediated regulation of stomatal guard cells. The CPK8 mutant was more sensitive to drought stress, while overexpressing CPK8 in transgenic plants exhibited tolerance [97,109]. *CaCPK1* activity increases the chickpea responsiveness to drought stress, and its activity is ubiquitous in all tissues of the plant [110]. The activation of drought-responsive CPK-encoding genes is also triggered by various biochemical pathways. A rice CPK-encoding gene (*OsCPK1*) specifically activated by sucrose starvation was involved in mechanism to prevent drought stress injury during germination by negatively regulating the expression of GA biosynthesis and activating the expression of a 14-3-3 protein ‘GF14c’ [111].

Some closely related CPK-encoding isoforms show functional diversity in response to drought stress. For example, functional divergence is present between two closely homologous (*TaCPK7* and *TaCPK12*) genes of wheat [112]. Functional analysis of *TaCPK7* and *TaCPK12* reveals that *TaCPK7* responded to H_2_O_2_, drought, salt, and low temperature, while T*aCPK12* responded only through the ABA signaling pathway [112]. Several transgenic studies have been conducted to characterize the functions of CPKs in different plant species in relation to drought stress response signaling in plants. The *ZoCDPK1* genes from ginger overexpressed in tobacco (*Nicotiana tabacum*) conferred drought as well as salinity tolerance by improving the photosynthesis and growth of the plant [113]. Enhanced expression of *ZoCDPK1* under drought and JA treatment was observed, but no variation was found in expression because of low-temperature stress and abscisic acid treatment. *ZoCDPK1* induces the expression of stress-responsive genes (i.e., early responsive to dehydration stress (*ERD1*) and responsive to dehydration (*RD21A*)). In ginger, it controls the stress signaling pathway and works in a CTR/DRE-independent manner [113]. Expression of CPK encoding genes of maize studied in *Arabidopsis* shows that *ZmCPK4* is involved in resistance to drought stress through ABA-regulated stomatal regulation. *ZmCPK4* induced by H_2_O_2_ and ABA treatment shows that there might be an association between mitogen-activated protein kinase (MAPKs) members and *ZmCPK4* in the upregulation of ABA-regulatory components, especially ABA-insensitive (ABI5), ABF3, and Ras-associated binding protein (RAB18) [87]. The functions of several drought-responsive CPK-encoding genes are summarized in Table 2. (Details of all the genes are given in Table S1)

### 4.2. CPKs-Mediated Salt Response Signaling

Salt stress is also a major abiotic factor limiting plant growth and global agricultural productivity. Salinity, mostly due to the accumulation of sodium Na^+^ and chloride Cl^−^ ions, causes an ion imbalance that leads the plants toward oxidative stress [152]. These ions also induce the toxicity of other ions in plants. Salts also increases the production of ROS in plants. Several studies have presented the functioning of CPK-encoding genes in plants against salt stresses. In *Arabidopsis*, *AtCPK27* genes were found in favor of plant adaptation against salt stress [125]. Disruption in the expression of *CPK27* in a T-DNA insertional mutant shows salt hypersensitivity at early growth stages in Arabidopsis. *CPK27* regulated H_2_O_2_ and ionic homeostasis. *AtCPK3* functions in guard cell movement through osmotic adjustment and ion channel regulation during salt accumulation [11,117,118]. The overexpression of AtCPK*3* also increases ABA sensitivity and salt hypersensitivity, affecting the seedling growth and stomatal regulation [98,117]. *AtCPK6* belongs to a subclass of the CPK gene family in *Arabidopsis* whose expression is induced under salt-stressed conditions. *AtCPK6* and other kinases are activated because of cytoplasmic Ca^2+^ elevation in the calcium-dependent pathway, which depends on ABA. These kinases combined with *AtCPK6* trigger the salt and osmotic stress tolerance. Overexpression of *AtCPK6* in *Arabidopsis* increases the drought and salt tolerance in transgenic plants. RT-PCR analyses showed an increase in the expression of salt-regulated genes in plants, in which the *AtCPK6* gene was over-expressed [119].

**Figure 2:**
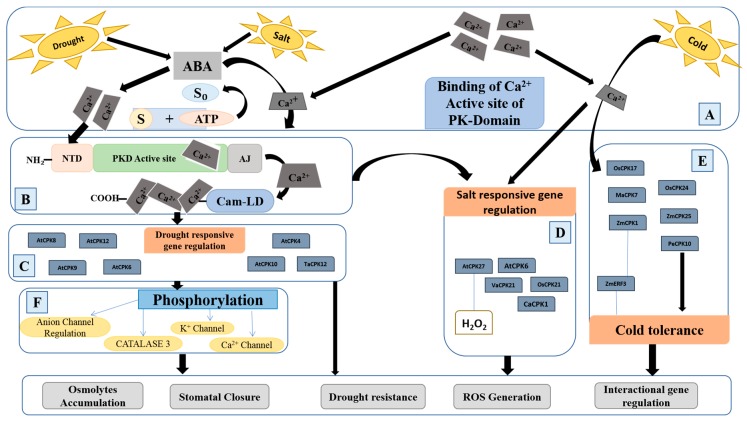
Role of different CPKs under various abiotic stresses; (**A**) Ca^2+^-dependent ABA-mediated drought and salt stress signal recognition by CPKs; (**B**) Ca^2+^ binding at the active site of protein kinase domain (PKD); (**C**) some drought-responsive genes involved in metabolite regulation and signal transduction pathways; (**D**) some salt-responsive genes and their role in antioxidant production (i.e., H_2_O_2_), as well as ROS detoxification; (**E**) some cold stress-responsive genes and their interaction genes activation; and (**F**) phosphorylation events controlling the anion channel regulation, K^+^-inward channel regulation, Ca^2+^-concentration, and channel regulation in the cell, and ABA-mediated CATALASE 3 regulation in plant cells.

*OsCPK12* positively modulates salt stress tolerance, and it is associated with decreases in the resistance against blast disease by increasing the sensitivity to ABA and inducing the accumulation of ROS in rice [1]. In *Arabidopsis*, *AtCPK27* was found to be favorable for plant adaptation against salt stress. Disruption in the expression of *CPK27* in T-DNA insertional mutant shows salt hypersensitivity at early growth stages. Under salt stress, *CPK27* regulates H_2_O_2_ and ionic homeostasis and makes plants resistant to salt stress (Figure 2) [125].

*OsCPK21* genes regulate the ABA-dependent salt stress signaling pathway. The high survival rate of transgenic rice seedlings developed by a mini scale, full-length cDNA over-expresser (FOX) gene hunting system was found due to the overexpression of *OsCPK21*-FOX under salt stress. In these plants, many salt-induced and ABA-regulating genes were expressed more as compared to wild-type plants. Overexpression of *OsCPK21* increases exogenous ABA and enhances salt tolerance by regulating and inducing the salt tolerance genes [136].

*VaCPK21* gene up-regulation is positively involved in salt stress-response signaling mechanisms in grapevines. Overexpression of this gene in transgenic *Arabidopsis* and *V. amurensis* callus cell lines shows that under the salt stress, *VaCPK21* acts as a regulator for genes that respond to salt stress (i.e., *AtRD26*, kinase-like protein (*AtKIN1), AtRD29B, AtNHX1*, catalase (*AtCAT1*), copper superoxide dismutase (*AtCSD1*), cold regulator (*AtCOR15* and *AtCOR15*)), and are found functionally important for salt stress tolerance [149]. Similarly, *CaCPK1* and *CaCPK2* activities are enhanced during high salt stress in leaves of chickpea plants. These isoforms play a role in the regulation of phytohormones and defense signaling pathways [110].

### 4.3. CPK-Dependent Cold and Heat Stress Signaling

Several CPK-encoding genes are differentially expressed under cold and heat treatments, but their exact molecular response mechanism is still unknown. *OsCPK17* was reported to be important for the cold stress response by targeting the sucrose synthase and plasma membrane intrinsic proteins in rice [135]. *OsCPK24* causes inhibition of glutaredoxin (OsGrx10) to sustain higher glutathione levels and phosphorylation, through the Ca^2+^ signaling pathway, and responds positively to cold stress tolerance in rice [74]. *MaCDPK7* was found as a positive regulator of heat-induced fruit ripening and chilling stress tolerance in bananas [146].

*PeCPK10* provides cold and drought stress tolerance through ABA-induced stomatal closing in *P. euphratica.* Its constitutive expression regulates ABA-responsive genes (i.e., *RD29B* and *COR15A*) that regulate the cellular functioning. Transgenic *Arabidopsis* with over-expressed *PeCPK10* showed lower water loss under drought stress and tolerance against freezing. Expression analyses reveal that *PeCPK10* localizes in cytoplasm quickly in response to changes in Ca^2+^ concentrations and regulates the stomata guard cells, while nuclear-localized *PeCPK10* only regulates the transcriptional factors [150]. *CPK16* and *CPK32* in grapevine plants positively regulate stilbene (a phenolic secondary metabolite) biosynthesis and CPK30 individually involved in both cold and drought tolerance [153]. In maize, *ZmCPK1* and *ZmCPK25* gene expressions were increased or decreased, respectively, upon exposure to cold stress. *ZmCPK1* is negatively related with the regulation of the cold stress signaling mechanism. Studies of transgenic *Arabidopsis* also show that *ZmCPK1* inversely regulates the expression of ethylene response factor (*ZmERF3*) genes and impairs cold stress tolerance [33]. *CsCDPK20* and *CsCDPK26* act as regulatory factors for heat stress-responsive genes and control positive heat stress signaling in the tea plant [144].

### 4.4. Role of CPKs in ROS Detoxification

Drought, salt, and heat stress triggers ROS production in plants, which must be detoxified by the plant to prevent itself from oxidative stress. Mitochondria, chloroplasts, and peroxisomes are the central organelles for ROS accumulation [105,154]. ABA-induced ROS production in plants is reported to be dependent on nicotinamide adenine dinucleotide phosphate hydrogen (NADPH) oxidase [105], which plays a vital role in oxidative bursting and activating plant defense responses [155,156]. Plant CPKs have been reported to regulate ROS production [2]. For instance, *StCPK4* functions in the phosphorylation of NADPH oxidase and indirectly regulates ROS accumulation [143]. In *B. napus*, *BnaCPK2* controls the activity of the respiratory burst oxidase homolog protein D (RbohD) during cell death and ROS production [2]. Arabidopsis *CPK32* interacts with ABF4 in the ABA signaling pathway [126]. *AtCPK6* from *Arabidopsis* decreases ROS production by reducing lipid peroxidation and confers drought stress [119]. Likewise, *OsCPK12* promotes salt stress tolerance in rice through decreasing ROS accumulation [1]. The other CPKs and ROS responses are summarized in Table 2.

## 5. Functional Interaction of CPKs with Other Kinases in Abiotic Stress Signaling

CPK crosstalk and several interactions have been revealed in molecular regulatory pathways by functional studies. CPKs are not only involved in specific stress responses but also in multiple stress-related pathways by interacting with other distant proteins and regulating phosphorylation events. In *Arabidopsis*, *CPK28* supports the turnover and phosphorylation of plasma membrane-related receptor-like cytoplasmic kinase (botrytis-induced kinase 1, BIK1), an important convergent substrate of multiple pattern recognition receptor (PRR) complexes for plant immunity [36]. *AtCPK8* regulates and phosphorylates CAT3. It is involved in Ca^2+^-dependent ABA and H_2_O_2_-induced guard cell regulation and provides drought resistance [97,109]. Molecular responses of *AtCPK1* studied by using real-time PCR (RT-PCR) show that the investigated gene expressions, viz., pyrroline-5-carboxylate synthetase 1(*P5CS1*), galactinol synthase 1(*GOLS1), RD22* (dehydration-responsive protein)*, RD29A*, C-repeat binding factor (CBF4), and *KIN2* (kinases), were upregulated by *ATCPK1* and conferred salinity stress tolerance [157]. Further, *AtCPK1* in loss-of-function and gain-of-function mutants were studied. It provides salt and drought stress resistance by up and down-regulation of stress responder genes, viz., zinc finger protein (*ZAT10*), *APX2*, *COR15A*, and *RD29A* [157]. *AtCPK12* phosphorylates several salt stress response-related proteins during regulatory functioning [72]. Another grapevine gene (*VaCPK21*) transgenically expressed in *Arabidopsis* interacts with several salt stress-related genes (i.e., *AtRD29*, *AtRD26*, *AtKIN1*, *AtNHX1*, *AtCSD1*, *AtCAT1*, *AtCOR15*, and *AtCOR47*). Likewise, *VaCPK20* responds to cold and drought stress tolerance by regulating *COR47*, *NHX1*, *KIN1*, or *ABF3* in transgenic *Arabidopsis* [107,149].

In vivo interaction validated by co-immunoprecipitation assays (Co-IP) revealed that *OsCPK4*, a dual-face protein, was involved in the regulation of the stability of cytoplasmic kinase (*CPK176*) in rice. *OsCPK4* plays a vital role in the negative regulation of receptor-like *OsCPK176* accumulation. *OsCPK4* and *OsCPK176* phosphorylation events provide pattern-triggered immunity [130]. *OsCPK17* phosphorylates the sucrose-phosphate synthase (*OsSPS4*) and plasma membrane intrinsic proteins (*OsPIP2;1* and *OsPIP2;6*) (aquaporin), which are essential in sugar metabolism and membrane channel activity against cold stress responses in rice [135]. Moreover, *OsCPK24* is involved in the phosphorylation of glutathione-dependent thioltransferase and inhibition of *OsGRX10* to maintain a higher level of glutathione. This regulatory pathway induces the overall cold stress responsiveness in rice [74]. The plant CPK-encoding genes also induce the regulation of other stress-responsive genes, viz., *AtRBOHF*, *AtRBOHD*, *AtABI1*, *AtRAB18*, *AtRD29B*, *AtHSP101*, *AtHSP70*, *Arabidopsis* heat stress transcription factor A2 (*AtHSFA2*), *AtP5CS2*, proline transporter (*AtProT1*), *AtPOD*, and *AtAPX1* for drought, salt, heat and cold stresses [11]. In tea plants, *CsCDPK20* and *CsCDPK26* have an interactive function for thermo-tolerance [144]. BnaCPK2 interacts with NADPH oxidase-like RbohD and controls ROS accumulation and cell death in oilseed rape [2]. In *Arabidopsis*, *CPK9* controls the ABA ion channel regulation through a Ca^2+^-dependent manner. Overexpression studies revealed that CPK9 and CPK33 mutually controlled the regulation of guard cells and stomatal movement [75]. *CPK16* and *CPK32* in grapevine plants positively regulate stilbene (a phenolic secondary metabolite) biosynthesis and *CPK30* individually involved in both drought and cold tolerance [153]. Moreover, *VaCPK1* and *VaCPK26* genes are also involved in the same regulatory pathway ([89]. The overexpression of VaCPK29 up-regulates stress-responsive genes (i.e., dehydration elements (DREs) *AtABF3*, *AtDREB1A*, *AtDREB2A*, *AtRD29A*, and *AtRD29B*), which provide resistance to heat as well as osmotic stress [73]. Under in vitro conditions, post-transcriptionally miR390-regulated *StCDPK1* controls the downstream auxin efflux carrier of PIN-proteins (*StPIN4*), which are involved in potato tuber development [142].

*Arabidopsis* CPKs interact and phosphorylate the basic leucine zipper domain (bZIP) transcription factor FD and have a crucial role in florigen complex formation, which induces late flowering in plants [127]. Biochemical analyses show that the cold-induced marker gene (*Zmerf3*), which is a type II ethylene response factor, is suppressed by *ZmCPK1* in maize. It is supposed that the *ZmCPK1* directly phosphorylates the ERF3 protein and, as a result, inactivates ERF and has a negative role in the cold stress response [33]. *ZmCPK11* controls the upstream *ZmMPK5*, which is involved in ABA-dependent defense-related signaling in maize. CPK-encoding genes also have several interactive functions concerning plant growth and development. In *Xenopus* oocytes, AtCPK32 potentially regulates the cyclic nucleotide-gated ion channel regulating gene (*CNGC18*). AtCPK32 stimulation of CNGC18 regulates pollen tube depolarization in *Arabidopsis* [83]. Constitutively active *OsCDPK1* in gain and loss-of-function transgenic rice targets the G-box factor 14-3-3c protein (GF14c). The expression of this protein causes the biosynthesis of GA and improves drought tolerance in rice seedlings [111]. AtCPK28 seems to be a regulatory component for the control of stem length and vascular development in *Arabidopsis*. The mutant of CPK28 (i.e., cpk28) was involved in the altered expression of NAC transcriptional regulators, such as NST1 and NST3, as well as gibberellin-3-beta-oxigenase 1 (GA3ox1), a regulator of gibberellic acid homeostasis [81]. After ABA treatment, the dual functioning *OsCPK9*-OX in rice increases the transcript levels of drought and spikelet fertility-responsive genes, viz., *OsRSUS*, *Rab21*, *Osbzip66*, and *OsNAC45*. The results confirmed by quantitative reverse transcription polymerase chain reaction (qRT-PCR) demonstrate that *OsCPK9* in interacting with these genes switches on the molecular regularization of ABA and stress-associated pathways [71]. The *ZoCDPK1* gene from ginger promotes the expression of drought and salinity stress associated genes, viz., RD2A (dehydration responsive protein 2A) and ERD1 (early responsive to dehydration stress 1) in tobacco. This DRE/CRT-independent regulatory pathway improves photosynthesis and plant growth as well [113]. Constitutive expression of calcium-dependent protein kinase of *Populus euphratica* (*PeCPK10*) regulates (*RD29B* and *COR15A*) cold and drought genes [150]. This cross-talk between CPK isoforms and the interactive partners increases the complexities among the signaling pathways.

## 6. Conclusions

The multifaceted role of CPKs in plants is consequential for abiotic stress tolerance in plants. Regardless of the reported functional detail on CPK-encoding genes, there are many other important isoforms identified whose expression profiles and involvement in abiotic stress signal transduction pathways in plants are still not clearly known. Future research is required to extend and identify the remaining CPK-encoding genes, their interactional regulators, and their functional exploration with respect to abiotic stress responses. These research studies are helpful to improve the plant’s adaptation under unpredictable environments and to minimize threats to the world’s food security.

## Figures and Tables

**Table 1:** Genome-wide identification of calcium-dependent protein kinases (CPKs) among various plant species.

Sr. #	Common Name	Botanical Name	No. of CPKs	Genome Size (Mb)	Reference
1	Algae	*Volvox carteri*	6	131.2	[37]
2	Apple	*Malus domestica*	28	881.3	[37]
3	Arabidopsis	*Arabidopsis thaliana*	34	135	[20]
4	Banana	*Musa acuminata*	44	523	[38]
5	Barley	*Hordeum vulgare*	27	667	[39]
6	Barley	*Hordeum vulgare*	28	667	[31]
7	Barrel clover	*Medicago truncatula*	11	360	[37]
8	Black cottonwood	*Populus trichocarpa*	28	422.9	[37]
9	Poplar	*Populus trichocarpa*	30	500	[34]
10	Butcher	*Micromonas pusilla*	22	2	[13,37]
11	Cacao tree	*Theobroma cacao*	17	346	[13,37]
12	Canola	*Brassica napus*	25	1130	[40]
13	Cassava	*Manihot esculenta*	26	532.5	[30]
14	Caster bean	*Ricinus communis*	15	400	[37]
15	Castor bean	*Ricinus communis*	15	400	[13,37]
16	Chinese liquorice	*Glycyrrhiza uralensis*	23	379	[41]
17	Chlamydomonas	*Chlamydomonas reinhardtii*	14	111.1	[13,37]
18	Clementine	*Citrus clememtina*	26	301.4	[37]
19	Cocoa tree	*Theobroma cacao*	17	346	[37]
20	Columbine	*Aquilegia coerulea*	16	306.5	[13,37]
21	Cotton	*Gossypium raimondii*	41	880	[28]
22	Cotton	*Gossypium hirsutum*	98	2250–2430	[42]
23	Cucumber	*Cucumis sativus*	19	323.99	[43]
24	Cucumber	*Cucumis sativus*	18	203	[37]
25	Finger Millet	*Eleusine coracana*	4	1593	[44]
26	Flax	*Linum usitatissimum*	47	318.3	[37]
27	Flooded gum	*Eucalyptus grandis*	22	691	[37]
28	Foxtail Millet	*Setaria italic*	27	405.7	[37]
29	Foxtail Millet	*Setaria italic*	29	405.7	[45]
30	Foxtail millet	*Setaria italica*	27	405.7	[13,37]
31	Grape	*Vitis vinifera*	19	500	[29]
32	Grapevine	*Vitis amurensis*	17	500	[46]
33	Grapevine	*Vitis amurensis*	13	500	[47]
34	Green algae	*Coccomyxa subellipsoidea*	2	49	[13,37]
35	Green algae	*Ostreococcus lucimarinus*	3	13.2	[13,37]
36	Green bean	*Phaseolus vulgaris*	25	521.1	[37]
37	Linseed	*Linum usitatissimum*	47	318.3	[13,37]
38	Maize	*Zea mays*	35	2500	[48]
39	Maize	*Zea mays*	40	2500	[49]
40	Maize	*Zea mays*	47	2500	[37]
41	Melon	*Cucumis melo*	18	375	[32]
42	Monkey flower	*Mimulus guttatus*	25	321.7	[37]
43	Mustard	*Brassica rapa*	49	283.8	[37]
44	Norway spruce	*Picea abies*	11	1960	[37]
45	Oilseed rape	*Brassica rapa*	49	283.8	[13,37]
46	Orange	*Citrus sinensis*	24	319	[13,37]
47	Papaya	*Carica papaya*	15	135	[13,37]
48	Papaya	*Carica papaya*	15	135	[37]
49	Peach	*Prunus persica*	17	227.3	[37]
50	Pepper	*Capsicum annuum*	31	407.5	[50]
51	Pigeon Pea	*Cajanus cajan*	23	852	[51]
52	Potato	*Solanum tubersum*	21	800	[37]
53	Potato	*Solanum tuberosum*	23	800	[52]
54	Purple false brome	*Brachypodium distachyon*	27	272	[37]
55	Purple false brome	*Brachipodium distachyon*	27	272	[37]
56	Red Shepherd’s Purse	*Capsella rubella*	32	134.8	[37]
57	Rice	*Oryza sativa*	29	430	[53]
58	Rice	*Oryza sativa*	22	430	[54]
59	Rice	*Oryza sativa*	30	372	[37]
60	Rubber tree	*Hevea brasiliensis*	30	1332	[55]
61	Salt cress	*Thellungiella halophile*	31	238.5	[13,37]
62	Shepherd’s Purse	*Capsella rubella*	32	134.8	[37]
63	Sorghum	*Sorghum bicolor*	28	697.5	[37]
64	Soybean	*Glycine max*	39	1115	[56]
65	Soybean	*Glycine max*	50	1115	[57]
66	Soybean	*Glycine max*	39	1115	[58]
67	Soybean	*Glycine max*	41	978	[13,37]
68	Spikemosses	*Selaginella moellendorffii*	11	212.5	[13,37]
69	Spreading earthmoss	*Physcomitrella patens*	25	480	[13,37]
70	Sweet orange	*Citrus sinensis*	24	319	[37]
71	Switchgrass	*Panicum virgatum*	53	1358	[37]
72	Tobacco	*Nicotiana tabacum*	15	323.75	[59]
73	Tomato	*Solanum lycopersicum*	29	900	[60]
74	Tomato	*Solanum lycopersicum*	28	900	[37]
75	Tomato	*Solanum lycopersicum*	29	900	[61]
76	Wheat	*Triticum aestivum*	20	2125	[26]
77	Wild Strawberry	*Fragaria vesca*	14	240	[37]

**Table 2:** Various functions of CPKs in biotic and abiotic stresses in different plant species.

Sr. #	Specie Name	Gene	Function	Reference
1	*Arabidopsis thaliana*	*AtCPK1*	Cellular homeostasis, resistance fungal elicitor.	[76,78,114,115,116]
2		*AtCPK3*	Salt resistance.	[117,118]
3		*AtCPK4*	Regulate ABA-regulatory transcription factors (e.g., ABF, ABF4, drought resistance).	[98]
4		*AtCPK5*	Regulate immunity responses, ROS-dependent cell-to-cell communication.	[78]
5		*AtCPK6*	Drought tolerance, ABA-dependent osmotic adjustment.	[119]
6		*AtCPK8*	Drought tolerance through interaction with protein CAT3.	[97,109]
7		*AtCPK9*	Regulate the ABA-dependent signaling pathway interacting with *CPK33.*	[75]
8		*AtCPK10*	Drought responsiveness, ABA-mediated stomatal movements.	[106]
9		*AtCPK11*	Phosphorylation of AtDi19, ABA signaling.	[120]
10		*AtCPK12*	Seed germination, activation of ABA regulators.	[72,121]
11		*AtCPK16*	Root-gravitropism phosphorylate *AtACS7*.	[122]
12		*AtCPK21*	Hyperosmotic adjustments.	[123]
13		*AtCPK23*	Salt stress, drought stress.	[124]
14		*AtCPK27*	Salinity resistance, H_2_O_2_ and ionic homeostasis.	[125]
15		*AtCPK28*	Vascular development, stem elongation, ethylene synthesis, lignin deposition.	[81,82]
16		*AtCPK32*	ABA-regulatory gene activation.	[126]
17		*AtCPK33*	Regulates flowering, biosynthesis of florigen and flowering locus T protein.	[127]
18	*Cicer areitinum* (Chickpea)	*CaCPK1*	Salt stress, drought stress, phytohormones, and defense signaling pathways.	[110]
19		*CaCPK2*		
20	*Capsicum annuum* (Peppers)	*CaCPK3*	Pathogen resistance, defense functioning (i.e., regulates jasmonic and salicylic acid), ethephon.	[79]
21	*Fragaria* x *ananassa* (Garden strawberry)	*FaCPK1*	low-temperature tolerance, fruit ripening.	[128]
22	*Medicago sativa* (Alfalfa)	*MsCPK3*	Heat stress resistance, embryogenesis.	[129]
23	*Oryza sativa* (Rice)	*OsCPK1*	Drought stress, seed germination, and GA biosynthesis.	[111]
24		*OsCPK4*	Microbial-associated immunity, OsRLCK176 degradation.	[130]
25		*OsCDPK5*	Fungal attacks phosphorylate OsERG1 and OsERG3.	[131]
26		*OsCPK9*	Drought stress tolerance, ABA sensitivity spikelet fertility.	[71]
27		*OsCPK10*	*Pseudomonas syringae pv* resistance, SA and JA regulator.	[132]
28		*OsCPK12*	Salt tolerance, blast disease resistance, induce ROS production, leaf senescence,	[1,133]
29		*OsCDPK13*	Regulate cold, salt, dehydration responses.	[134]
30		*OsCPK17*	Cold stress interacts with sucrose synthase and plasma membrane intrinsic proteins.	[135]
31		*OsCPK21*	Salt tolerance, ABA pathway activation.	[136]
32		*OsCPK24*	Cold stress tolerance, inhibition of OsGrx10.	[74]
33		*OsCPK31*	Starch accumulation, early grain filling.	[137]
34	*Nicotiana tabacum* (Tobacco)	*NtCPK1*	Signaling localization for repression of shoot growth, GA biosynthesis.	[138]
35		*NtCPK2*	Biotic stress immunity.	[139]
36		*NtCPK32*	Pollen tube growth interacts with CNGC18.	[83]
37	*Hevea brasiliensis* (Rubber tree)	*HbCDPK1*	Latex biosynthesis, rubber production.	[84]
38	*Panax ginseng* (Chinese ginseng)	*PgCDPK1a*	Regulate ginseng growth.	[85]
39	*Phalaenopsis amabilis* (Moth orchid)	*PaCPK1*	Cold stress sensitivity, wounding, pathogen attack.	[86]
40	*Triticum aestivum* (Wheat)	*TaCDPK1*	Regulate metabolic and developmental pathways.	[91]
41		*TaCPK7*	Drought stress, salt stress, ABA signaling pathway.	[112]
42		*TaCPK12*		
43	*Zingiber officinale* (Ginger)	*ZoCDPK1*	Salinity and drought stress tolerance.	[113]
44	*Zea mays* (Maize)	*ZmCPK1*	Cold stress regulates ZmERF3 expression.	[33]
45		*ZmCPK4*	Upregulate ABA-regulatory components (i.e., ABI5, ABF3 and RAB18) with MAPKs.	[87]
46		*ZmCPK11*	Superoxide dismutase and ascorbate peroxidase production, ABA pathway.	[103]
47	*Vigna radiata* (Mung bean)	*VrCPK1*	Salt stress tolerance.	[140]
48	*Vicia faba* (Broad bean)	*VfCPK1*	Drought stress resistance.	[108]
49	*Solanum lycopersicum* (Tomato)	*SlCDPK2*	Flowering.	[141]
50		*SlCDPK10*	*Xanthomonas oryzae* pv. *oryzae* and *Pseudomonas syringae* resistance.	
51		*SlCDPK18*	*Xanthomonas oryzae* pv. *oryzae* and *Pseudomonas syringae* resistance.	[60]
52	*Solanum tuberosum* (Potato)	*StCPK1*	Tuber formation.	[142]
53		*StCPK4*	Fungal pathogen resistance, ROS production.	[143]
54		*StCDPK5*	Blight resistance and susceptibility, ROS defense functioning.	[100]
55		*StCDPK7*	Resistance against *Phytophthora infestans*.	[77]
56	*Nicotiana attenuate* (Coyote tobacco)	*NaCDPK4*	Wound-induced jasmonic acid (JA) accumulation, insect resistance.	[80]
57		*NaCDPK5*		
58	*Camellia sinensis* (Tea plant)	*CsCDPK20*	High-temperature stress resistance.	[144]
59		*CsCDPK26*		
60	*Hordeum vulgare* (Barley)	*HvCPK3*	Resistance against powdery mildew.	[145]
61		*HvCPK4*		
62	*Brassica napus* (Oilseed rape)	*BnaCPK2*	ROS accumulation, cell death.	[2]
63	*Musa acuminate* (Banana)	*MaCDPK7*	Heat-induced fruit ripening, chilling, stress tolerance.	[146]
64		*MaCDPK2*	Sensitive to Foc-TR4 infection, biotic stress tolerance.	[147]
65		*MaCDPK4*	Sensitive to Foc-TR4 infection, biotic stress tolerance.	
66		*MaCDPK3*	Responsive for drought, cold, and salinity.	
67	*Vitis amurensis* (Grapevine)	*VaCPK1*	Salt stress, heat-responsiveness, stilbene bio-synthesis.	[89,148]
68		*VaCPK26*	Salt stress, Stilbene bio-synthesis, through the induced expression of stilbene synthase (STS) genes.	[89,148]
69		*VaCPK20*	Drought stress, cold stress.	[107]
70		*VaCPK21*	Salt stress signaling.	[149]
71	*Pharbitis nil* (Picotee)	*PnCPK1*	Seed germination, seedling growth, flowering, regulation of light-dependent pathways, embryogenesis.	[90]
72	*Populus euphratica* (Desert poplar)	*PeCPK10*	Drought and cold stress tolerance, ABA-responsive genes regulator.	[150]
73	*Cucumis melo* (Hami melon)	*HmCDPK2*	Resistance against Penicillium infection.	[151]

## Supplementary Materials

Supplementary materials can be found at https://www.mdpi.com/1422-0067/20/21/5298/s1.

## Abbreviations

ABA	Abscisic acid
AJ	Autoinhibitory junction
APX	Ascorbate peroxidase
AtABI1	*Arabidopsis thaliana* ABA-insenstive-1
AtCDPK/AtCPK	*Arabidopsis thaliana*-calcium dependent protein
AtCSD1	*Arabidopsis thaliana* copper superoxide dismutase 1
AtHSP101 and 70	*Arabidopsis thaliana* heat shock protein 101 and 70
AtProT1	*Arabidopsis thaliana* proline transporter 1
AtRBOHD	*Arabidopsis thaliana* respiratory burst oxidase protein D
AtRBOHF	*Arabidopsis thaliana* respiratory burst oxidase protein F
BaCDPK/BaCPK	*Brassica napus* calcium dependent protein kinase
BIK1	Botrytis-induced kinase 1
bZIP	Basic leucine zipper domain
Ca	Calcium
Ca2+	Calcium ion
CaCDPK/CaCPK	*Cicer arietinum* calcium dependent protein kinase
CaMs	Calmodulins
CAT	Catalase
CAT3	Catalase-3
CBD	Calcium binding domain
CBF4	C-repeat binding factor 4
CBLs	Calcineurin β-like proteins
CCaMK	Calcium/Calmodulin-dependent protein kinase
CDPKs/CPKs	Calcium dependent protein kinases
CMLs	Calmodulin-like protein Kinase
CNGC18	Cyclic nucleotide-gated ion channel 18
Co-IP	Co-immunoprecipitation assay
COR	Cold regulator
CT	C-terminus
CTR	C-repeat
DRE	Dehydration elements
EF	Elongation Factor
ERD1	Early responsive to dehydration stress 1
ERF3	Ethylene response factor 3
FaCDPK/FaCPK	*Fragaria* x *ananassa* calcium dependent protein kinase
GA	Gibberellic acid
GA3ox1	Gibberellin-3-betaoxigenase 1
GOLS1	Galactinol synthase 1
GPX	Glutathione peroxidase
GR	Glutathione reductase
H_2_O_2_	Hydrogen peroxide
HSF	Heat stress transcription factor
HSP	Heat shock protein
HvCDPK/HvCPK	*Hordeum vulgare* calcium dependent protein kinase
JA	Jasmonic acid
K^+^	Potassium ion
LeCDPK/LeCPK	*Solanum lycopersicum* calcium dependent protein kinase
MaCDPK/MaCPK	*Musa acuminate* calcium dependent protein kinase
MPK5	Mitogen-activated protein kinase 5
MsCPK	*Medicago sativa* calcium dependent protein kinase
NaCDPK/NaCPK	*Nicotiana attenuate* calcium dependent protein kinase
NADPH	Nicotinamide Adenine Dinucleotide Phosphate Hydrogen
NHX	Sodium/Hydrogen exchanger
NST	NAC-transcription factors
NtCDPK/NtCPK	*Nicotiana tabacum* calcium dependent protein kinase
N-VD	N-terminus variable domain
OsCPK/OsCDPK	*Oryza sativa* calcium dependent protein kinase
OsGrx10	*Oryza sativa* glutaredoxin 10
OX	Overexpression
P5CS1	Pyrroline-5-carboxylate synthetase 1
PaCDPK/PaCPK	*Phalaenopsis amabilis* calcium dependent protein kinase
PeCDPK/PeCPK	*Populus euphratica* calcium dependent protein kinase
PEG	Polyethylene glycol
PEST	Proline, glutamine, serine and threonine
PgCDPK/PgCPK	*Panax ginseng* calcium dependent protein kinase
PIP	Plasma membrane intrinsic protein
PKD	Protein kinase domain
PnCDPK/PnCPK	*Populus euphratica* calcium dependent protein kinase
PRR	Pattern recognition receptor
qRT-PCR	Quantitative reverse transcription Polymerase chain reaction
RAB18	Ras-associated binding protein 18
RbohD	Respiratory burst oxidase homolog protein D
RD2A	Dehydration responsive protein 2A
RD29A	Dehydration responsive protein 29A
ROS	Reactive oxygen species
RT-PCR	Real-time PCR
SiCDPK/SiCPK	*Setaria italic* calcium dependent protein kinase
SOD	Superoxide dismutase
StCDPK/StCPK	*Solanum tuberosum* calcium dependent protein kinase
TaCDPK/TaCPK	*Triticum aestivum* calcium dependent protein kinase
T-DNA	Transfer DNA
VaCDPK/VaCPK	*Vitis amurensis* calcium dependent protein kinase
VaCPK/VaCDPK	*Vitis amurenssis* calcium dependent protein kinase
VfCPK/VfCDPK	*Vicia faba* calcium dependent protein kinase
VrCDPK/VrCPK	*Vigna radiata* calcium dependent protein kinase
ZmCDPK1/ZmCPK1	*Zea mays* calcium dependent protein kinase 1
ZoCDPK/ZoCPK	*Zingiber officinale* calcium dependent protein kinase
